# Fine-tuning the performance of ddRAD-seq in the peach genome

**DOI:** 10.1038/s41598-021-85815-0

**Published:** 2021-03-18

**Authors:** Maximiliano Martín Aballay, Natalia Cristina Aguirre, Carla Valeria Filippi, Gabriel Hugo Valentini, Gerardo Sánchez

**Affiliations:** 1 Laboratorio de Biotecnología, Estación Experimental Agropecuaria (EEA) San Pedro, INTA, 2930 San Pedro, Argentina; 2 Instituto de Agrobiotecnología y Biología Molecular–IABiMo–INTA-CONICET, Instituto de Biotecnología, Centro de Investigaciones en Ciencias Veterinarias y Agronómicas, INTA, 1686 Hurlingham, Argentina

**Keywords:** Plant sciences, Genomics, Sequencing, Genetics, Genetic markers, Genomics, Plant breeding, Sequencing

## Abstract

The advance of Next Generation Sequencing (NGS) technologies allows high-throughput genotyping at a reasonable cost, although, in the case of peach, this technology has been scarcely developed. To date, only a standard Genotyping by Sequencing approach (GBS), based on a single restriction with ApeKI to reduce genome complexity, has been applied in peach. In this work, we assessed the performance of the double-digest RADseq approach (ddRADseq), by testing 6 double restrictions with the restriction profile generated with ApeKI. The enzyme pair PstI/MboI retained the highest number of loci in concordance with the in silico analysis. Under this condition, the analysis of a diverse germplasm collection (191 peach genotypes) yielded 200,759,000 paired-end (2 × 250 bp) reads that allowed the identification of 113,411 SNP, 13,661 InDel and 2133 SSR. We take advantage of a wide sample set to describe technical scope of the platform. The novel platform presented here represents a useful tool for genomic-based breeding for peach.

## Introduction

Peach (*Prunus persica* L. Batch) is the eighth most globally important fruit tree crop regarding world production^1^. The peach tree requires adequate winter chill to produce economically viable yields and therefore is mainly grown in temperate climates. Climate change is decreasing winter chill in areas where peach is traditionally cultivated, thereby threatening the production. Improving the performance of peach varieties to face climate changes requires multiple approaches and genomics may aid to this purpose^2^. The peach has a diploid small genome (230 Mbp) that was sequenced^3,4^. In addition several studies have reported the genetic control of important traits, including thermal requirement, which makes genomic selections a feasible approach for peach breeding (review in Gogorcena et al.^2^). On the other hand, the accomplishment of variation identification and genotyping requires high-throughput platforms. The first high-throughput genotyping platform developed was the 9K SNP Infinium II array v1^5^, an array composed of a prefixed set of 8144 SNPs covering the eight chromosomes of peach. This platform boosted the genomics studies allowing a deeper understanding of germplasm diversity, the construction of dense genetic maps for QTL analyses and Genome-Wide Association Study (GWAS) in peach. Several groups have taken advantage of the 9K SNP Infinium II array to construct highly density maps^6–10^, although in some cases the analyses do not cover all the chromosome^11–13^. The lack of polymorphic markers could be due to identity-by-descendant or an assortment bias of the chip. A study on 1576 peach accession showed that the proportion of SNP with low Minor Allele Frequency (MAF) was higher in a group of varieties of oriental origin^14^ that were not represented in the set of genotypes re-sequenced to construct the array^5^. These results could indicate that the SNPs most frequently present in commercial peach varieties may be overrepresented in the array. Indeed, the detection of variants with some degree of uniqueness in a given germplasm requires the availability of extensive sequencing data. The whole genome sequencing of germplasm collections of peaches and wild relative species allowed the identification of around 4 million of SNPs useful to study domestication^15^ and perform GWAS^16^ at a genome level.

The high cost of whole genome sequencing encourages researchers to use an intermediate approach that generate own genomic data at a reasonable cost. In this sense, RADseq has emerged as an alternative that takes advantage of NGS technologies by analyzing a small portion of the genome, while allowing multiplexing a large number of individuals on a sequencing lane. Nevertheless, in the case of peach, this approach remains poorly used. Several methods (RADseq, ddRADseq, GBS, MSG, among others) with minor modifications belong to “RADseq” or “GBS” (reviewed in Andrews et al.^17^). In general, these techniques consist of DNA libraries generated using one or two restriction enzymes and whose sequencing requires adapter ligation. Moreover, each library is tagged with unique barcode, which allows the in silico identification after sequencing. For example, a one restriction (with ApeKI enzyme) GBS platform originally developed for maize^18^ was applied in peach to genotype a F2 population^19^ and a germplasm collection^20^. Recently, a study has reported the use of a GBS-derived strategy, based on double restriction, to analyze interspecific hybrid used as *Prunus* rootstock^21^. In both cases, the researchers used the platforms to identify and genotype SNPs. To our knowledge, however, no other variant, such as InDel or SSR, has been detected by high throughput platform in peaches.

In this work, we present a novel ddRADseq genotyping platform for peach that involves two step: a double enzyme restriction digestion followed by a size selection step. A first analysis evaluating and comparing the results obtained by six double restriction digestions with those from the single one generated with ApeKI revealed that the combination of PstI/MboI was suitable for this species. After fine-tuning the conditions, a germplasm collection composed of 192 accessions (191 peaches and a plum) was genotyped. The study presents the methodological scopes of the platform and suggests ways to overcome technical limitations based on different experimental conditions used along the assay. Moreover, the analysis pipeline described could be useful for other RADseq or GBS approaches to discover experimental bottlenecks. The present platform allowed the identification of more than 125 K polymorphic variants of peach, including InDel and SSR, being a novel tool for genomic assisted breeding of this crop.

## Materials and methods

### Plant material and DNA extraction

The sample set consisted of 194 accessions from the germplasm active collection of San Pedro Research Station (San Pedro, INTA, Argentina) composed as follows: 190 *Prunus persica*, 3 rootstocks (*Prunus persica* background with contributions of *Prunus davidiana*) and 1 *Prunus salicina* (Supplementary Table S1). GHV is in charge of the peach active collection and performed the plant material identification and characterization. This collection belongs to the National Genetic Resources Network of INTA and is in agreement with national legislations. Fresh leaves from the selected trees were used for genomic DNA extraction with three extraction methods: CTAB (Cetyl Trimethyl Ammonium Bromide) method^22^, DNeasy plant Minikit protocol (Qiagen GmbH, Hilden, Germany) and NucleoSpin plant II kit protocol (Macherey–Nagel, Düren, Germany) according to the manufacturer’s recommendations. The DNA quality was verified by agarose gel electrophoresis analysis (Supplementary Fig. S1) and DNA quantification was carried out with Qubit dsDNA BR Assay Kit using a Qubit fluorometer (Thermo Fisher Scientific, Waltham, MA, USA) according to the manufacturer’s instructions.

### Evaluation of enzymes and size selection range

The performance of six different enzyme pair combinations (SphI/MspI; SphI/MboI; EcoRI/MspI, EcoRI/MboI; PstI/MspI and PstI/MboI) was tested in vitro and in silico. In each double digestion, one rare cutter (i.e. 6 bp recognition site: SphI, EcoRI and PstI) and one frequent cutter (i.e. 4 pb recognition site: MspI, MboI) were used. In addition, enzymes were selected to account for at least one methylation sensitive enzyme in the digestion pair, in order to avoid repetitive region sampling. The performance of the ApeKI restriction enzyme, used in reported GBS protocols for the species^19,20^, was also evaluated for comparison purposes. The digestions were performed as described previously by Aguirre et al.^23^.

In silico digestions were tested using the R package simRAD^24^. The *Prunus persica* reference genome (v2, accession number GCF_000346465.2) was retrieved from NCBI (https://www.ncbi.nlm.nih.gov/genome/annotation_euk/Prunus_persica/100/). The simulations were done following the proposal of Aguirre et al.^23^, whereas the digestion pattern plots were generated using a custom R script. The best enzyme pair combination for the species was defined as the one that generates the highest number of AB + BA fragments (i.e. fragments predicted to be generated by simultaneous digestion of both restriction enzymes) in the size selection range. The size selection range was kept between 300 and 400 bp, following Aguirre et al.^23^ recommendations.

### Library construction

Libraries were constructed essentially as described by Aguirre et al.^23^ at the Genomic Unit at IABiMo INTA-CONICET (Argentina). In brief, a ddRADseq derived protocol was optimized and applied on two samples (experiment 1) and subsequently scaled up to another 192 samples (experiment 2). PstI and MboI restriction enzymes were used to digest 150 ng of each gDNA at 37 °C for 90 min. The reaction was inactivated at 65 °C for 20 min and purified with 1.5 volumes of Ampure XP beads (Beckman Coulter, Brea, CA, USA).

The DNA fragments from the two samples in experiment 1 were ligated with the same universal adapters (A1 and A2) used in Aguirre et al.^23^, with the corresponding sticky-end modification to be complementary to the cutting pattern generated by each of the restriction enzymes tested here. The ligation was done using 2 pmol and 5 pmol of A1 and A2, respectively, and 2.4 Weiss units of T4 DNA ligase (Invitrogen, Carlsbad, CA, USA). The reaction was incubated for 1 h at 23 °C, followed by an additional incubation for 1 h at 20 °C. The inactivation of the reaction was performed at 65 °C for 20 min and the DNA was purified with 1 × Ampure XP bead per sample. A PCR was performed per sample with primers containing a pair of indexes. These primers, designed by Lange et al.^25^, have a portion for sequencing on the Illumina platforms plus an index (8 bp), which allows the identification of each library. NEB Phusion High-Fidelity DNA polymerase was used for PCR amplification, with the following cycling parameters: 3 min of initial denaturation (95 °C), 10 cycles of amplification (30 s at 95 °C, 30 s at 60 °C, 45 s at 72 °C), and 2 min of a final extension (72 °C). A 1.2× Ampure XP bead purification per PCR was performed after the amplification. The libraries were mixed by equal DNA quantity in one pool and concentrated in a SpeedVac (Eppendorf, Hamburg, Germany). Size selection was applied manually (in a range between 450 and 550 bp, corresponding to fragments of 410 to 510 bp length, when eliminating the adapters) through low-melting 1.5% agarose gel electrophoresis (Bio-Rad Laboratories, Hercules, CA, USA). The selected fragments were purified from the gel with QIAquick Gel Extraction kit (Qiagen N.V., Hilden, Germany).

The construction of the libraries for experiment 2 were performed by the service provided by Genomic Unit at IABiMo INTA-CONICET (Argentina) by ligating the DNA fragments from the 192 samples with 24 adapters barcoded (which were designed by Poland et al.^26^) under the same conditions of ligation as the experiment 1. After the ligation step, a check was performed by selecting random samples for qubit quantification and fragment analysis. The ligations were mixed by equal DNA quantity in 8 pools of 24 samples (with 24 different barcodes), then concentrated in a SpeedVac (Eppendorf, Hamburg, Germany) and finally cleaned by 1× Ampure XP bead purification per pool. An automatic size selection run was performed in a 2% agarose cassette in the SAGE ELF (Sage Science, Inc., Beverly, MA, USA) and the fragments of 450 bp on average (between 415 and 485 bp) were collected from one well. Subsequently, an extra step of 0.8× Ampure XP bead purification was performed to ensure the elimination of the fragments below 300 bp. A PCR was performed per pool of libraries with indexed primers identifying each pool, using the same conditions of PCR from the experiment 1.

The final libraries obtained in experiments 1 and 2 were quantified by Qubit fluorometer (Thermo Fisher Scientific, Waltham, MA, USA) and their quality was checked on a Fragment Analyzer system (Agilent Technologies, Santa Clara, CA, USA).

### Sequencing and data processing

The DNA libraries of Experiment 1 and Experiment 2 were paired-end sequenced (2 × 250 bp), with Illumina MiSeq (Experiment 1, at Genomic Unit, IABiMo INTA-CONICET, Argentina) and HiSeq 1500 technologies (Experiment 2 at INDEAR, Argentina). The quality and size of reads were verified with FASTQC^27^. The program process_radtag.pl of STACKS v2.0 software^28^ was used to filter the reads with uncalled bases, absence of enzyme recognition sites, presence of adapter sequence and low average Phred score (lower than 10). The reads were trimmed to 225 bases (because of the quality decrease in the last 25 bases; data not shown) and demultiplexed according to the specific barcodes. The filtered and trimmed sequences were aligned to the peach reference genome v2.0^4^ with BOWTIE2^29^ using default parameters and MAPQ > 3.

For each sample, the breadth and depth of coverage were determined with the utility “depth” of SAMtools package^30^ and two different Unix scripts. The alignment files from all samples were merged, thus producing a single file, with the utility “merge” of SAMtools package^30^. The coverage of the merged file was determined in the same way as in the individual samples. The reads count in 1000-bp bins, pairwise correlations, and heatmap was performed with Deeptools package^31^.

Principal Components Analysis (PCA) was performed and plotted with the R package PCAtools^32^. The number of common sites within each pool was detected using the SAMtools "depth" utility and counting all covered positions at least once in all samples of the analyzed pool with a Unix script. The artificial pools were created taking three samples from each experimental pool and organizing them in a new group as is described in Supplementary Table S1.

### Variant calling

The pipeline ref_map.pl (default parameters) of STACKS v2.0 software^28^ was used to call SNPs using the Bayesian genotype caller, which identifies the presence of an SNP within a locus by examining the read data from the entire metapopulation. The pipeline Population of STACKS v2.0 software^28^ was used to export the detected SNPs in VCF format. InDels were assessed using the package BCFtools^33^ with the multiallelic model and the detected InDels were analyzed with the MISA Perl script^34^ for the identification of SSRs. The MISA software analysis was performed with default parameters and only considering SSRs with motifs between one and six nucleotides in size. The minimum length was defined as ten repeat units for mononucleotides, six repeat units for dinucleotides and five repeat units for tri, tetra, penta and hexanucleotides. Finally, all the detected variants were stored in a VCF file. Prediction of variant effects was performed using the software SnpEff v4.3t^35^ (default parameters) and the gene annotation of the Peach genome v2.1 (https://www.rosaceae.org/species/prunus_persica/genome_v2.0.a1).

## Results

### Genome complexity reduction achieved with a double restriction digestion

The genome complexity reduction consisted of digestions using a combination between three rare cutter enzymes (PstI, SphI y EcoRI) and two frequent cutter enzymes (MboI y MspI) and by comparing the results with a single restriction reaction (with ApeKI). According to in silico simulation of restriction digestion of the complete peach genome, the PstI/MboI combination would produce the highest number of loci (63,730 loci) within the selected size values (300–400 bp) in relation to the other enzyme pair combinations (Supplementary Table S2). Moreover, PstI/MboI performed better than the ApeKI restriction digestion, within a range between 300 and 800 bp (Supplementary Table S2). That range corresponds to the one obtained in the regular GBS protocols, which do not perform a direct size selection, as ddRADseq do. Therefore, an indirect size selection using short PCR amplification cycles added to purification with low concentrations of Ampure Beads XP could be considered^23,36^.

**Figure 1:**
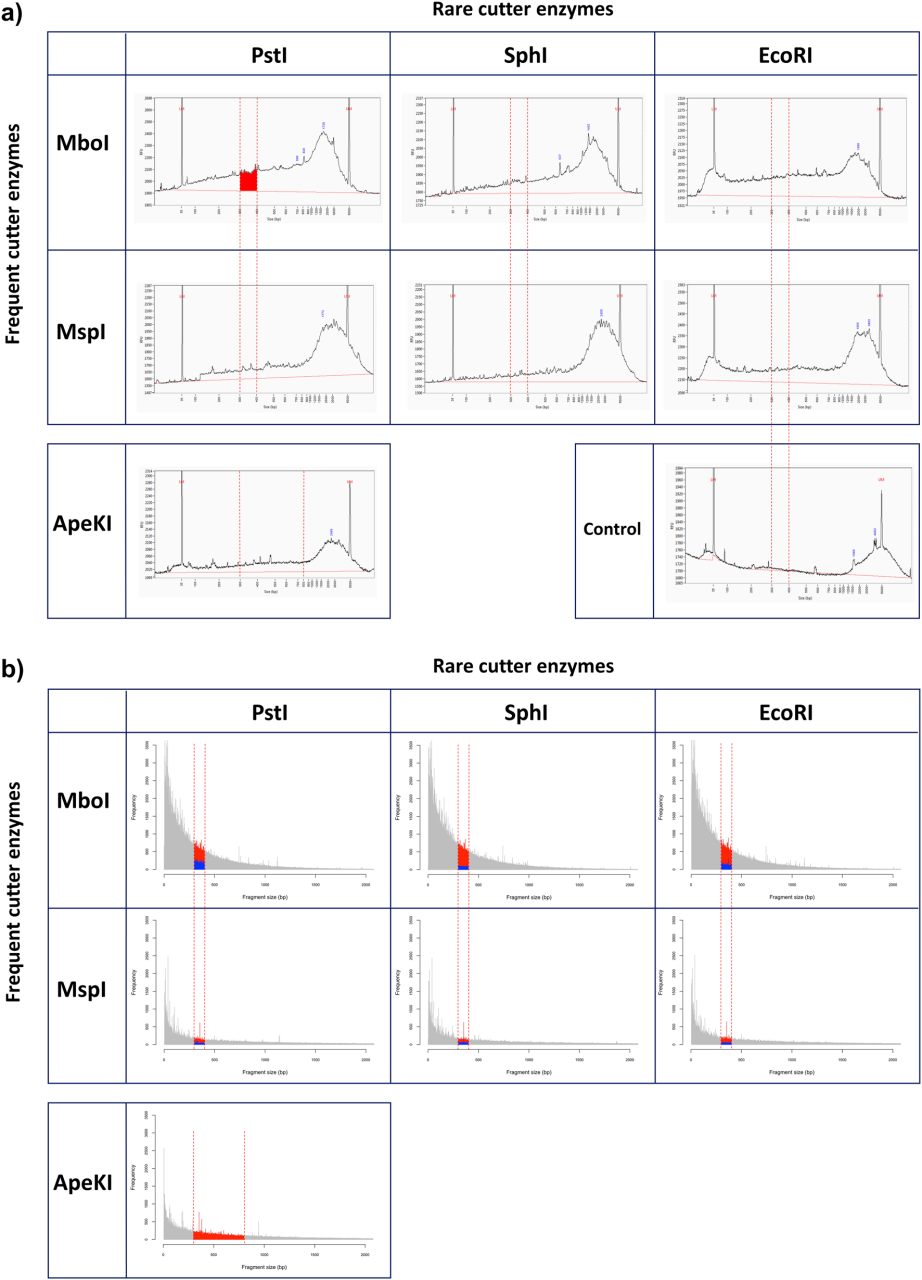
Reduction of genome complexity. (**a**) In vitro enzymatic restrictions. Profile of peach DNA (Dixiland) quantification by fragment analyzer (Agilent). The vertical red dashed lines indicate the region to be selected (300–400 pb for double restrictions and 300–800 pb for restriction generated by ApeKI). The larger area at the region to be selected (highlighted in red) was obtained for the combination of PstI/MboI. (**b**) In silico simulation of enzymatic restriction. Profile of the predicted restriction fragments generated using different enzyme pair combinations in the peach reference genome (v2.0). Grey area: all the restriction fragments generated by in silico digestion using one enzyme pair. Red area: fragments predicted in the range 300–400 bp. Blue area: AB + BA fragments (i.e. fragments predicted to be generated by simultaneous digestion of both restriction enzymes) in the range 300–400 bp.

In silico simulations were in accordance with the in vitro digestions, where PstI/MboI digestion retained the most abundant fragment population in the 300–400 bp region (Fig. 1). A preliminary estimation of the methodology by sequencing libraries from two parental of our breeding program, Dixiland and Summerprince, yielded 780,647 and 829,004 pair-end reads (2 × 250 pb), respectively (Experiment 1). An initial analysis identified 1437, 225 and 149 polymorphic and segregant SNP, InDel and SSR, respectively; which covered the 8 chromosomes of peach (data not showed). The comparisons of our results with data from previous studies was not possible, since the number of polymorphic markers between two genotypes depends on the analyzed genotypes as well as on the power of the platform. Nevertheless, the number of SNP were in the same order of the previous work that used GBS for genotyping a F2 population^19^. Therefore we considered that the experimental conditions were suitable and scaled up the protocol for the genotyping the whole germplasm collection (Experiment 2).

### Analysis of genome coverage of the platform

In experiment 2, a plum cultivar and 191 accessions (189 peaches and 3 rootstocks) from the EEA San Pedro germplasm active collection were analyzed (Supplementary Table S1). The libraries construction in batch of 24 samples, performed as described previously^23^, resulted in 8 pools. The DNA content of each pool was combined and normalized according to the DNA quantity for subsequent sequencing.

**Figure 2:**
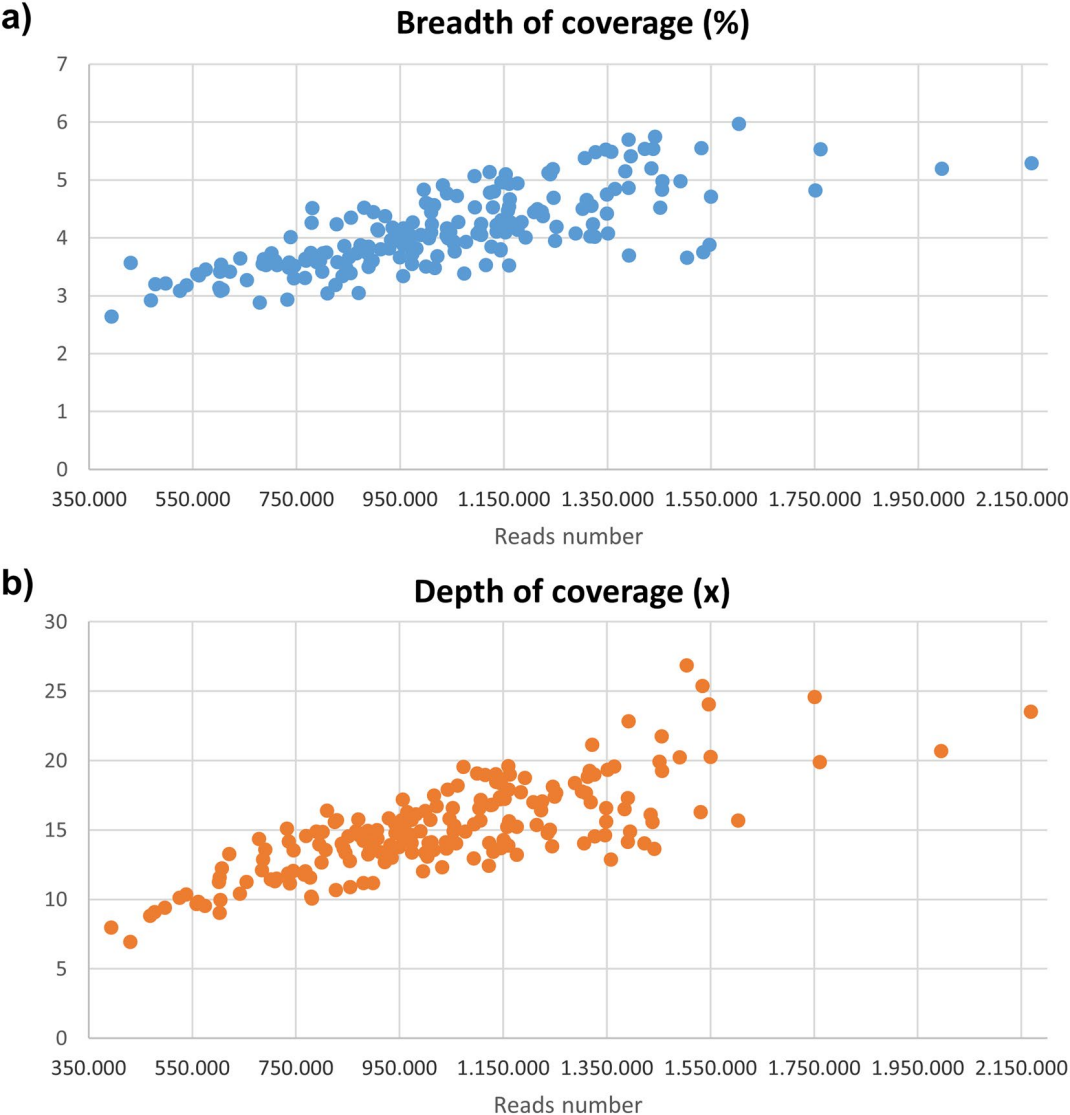
Increase of genome coverage by sequencing yield. The breadth (**a**) and depth (**b**) of coverages are shown.

The sequencing retrieved 200,759,000 of paired-end (2 × 250 bp) reads and after quality filtering, 98.3% of these reads were retained. Two samples: ‘Suncrest’ and ‘Flordaglobe’ retained hardly any reads (296 and 37,469, respectively) and, therefore, were discarded for further analyses. In average, the analysis of each sample retrieved 1.04 × 10^6^ paired-end reads (2 × 250 pb) with a variation coefficient (VC) of 28.14%. The reads obtained per sample were between 393,149 to 2,168,460, thus accumulating around 1 M reads (Supplementary Fig. S2). No significant differences were observed between total reads obtained from DNA extracted by CTAB method or commercial DNA extraction kits (data not showed). As expected, the higher the read number, the higher the breadth and depth coverage will result (Fig. 2). At around 1.5 × 10^6^ reads, the breadth coverage seems to reach a steady state of 5% with a minimum depth of 15×. The alignment of all data merged like a single individual gave a total coverage of the peach genome of 25%, with a mean depth of 15× (ranging from 7× to 27×).

**Figure 3:**
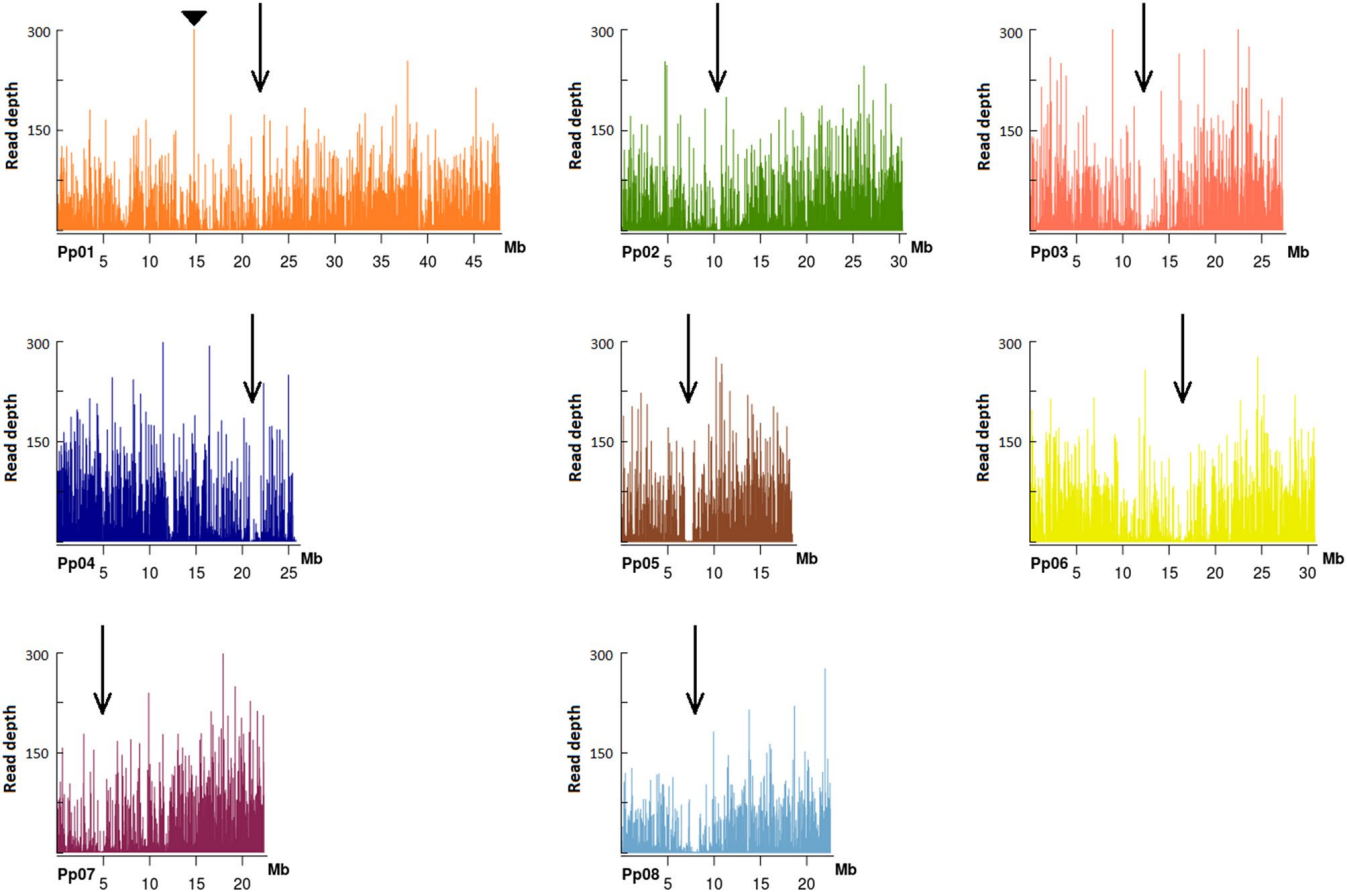
Reads distribution along the peach genome. The black arrows indicate the position predicted for the centromere according to Verde et al. ^4^. For chromosome 1 (Pp01), the read number scale is restricted to 300 for comparison proposes. Only the bin Pp01-14,777,945–14,778,945 (indicated with a triangle) showed more than 300 reads. Supplementary Fig. S3 displays the full scale graph.

The peach genome v2.0^4^ was separated in bins of 1000 bp and the reads obtained from each sample were mapped into bins to analyze the overlapping coverage. Although is possible that two or more reads (of 250 bp) found in a bin (of 1000 bp) could not actually overlap, we consider that bin size to simplify computation requirements. Therefore, the results of the analysis were taken as an estimation of actual common coverage between samples. As a result, the reads were evenly distributed around the eight chromosomes with the exception of the regions predicted to harbor the centromeres (Fig. 3). Most of the bins had less than 300 reads in average for each genotype. Surprisingly, a bin on chromosome 1 at positions 14,777,945–14,778,945 (Pp01-14,777,945–14,778,945) accumulated 10× more reads (2698) than the average (Supplementary Fig. S3). A blast analysis of the sequence of peach genome at that region showed high homology with mitochondrion sequences.

**Figure 4:**
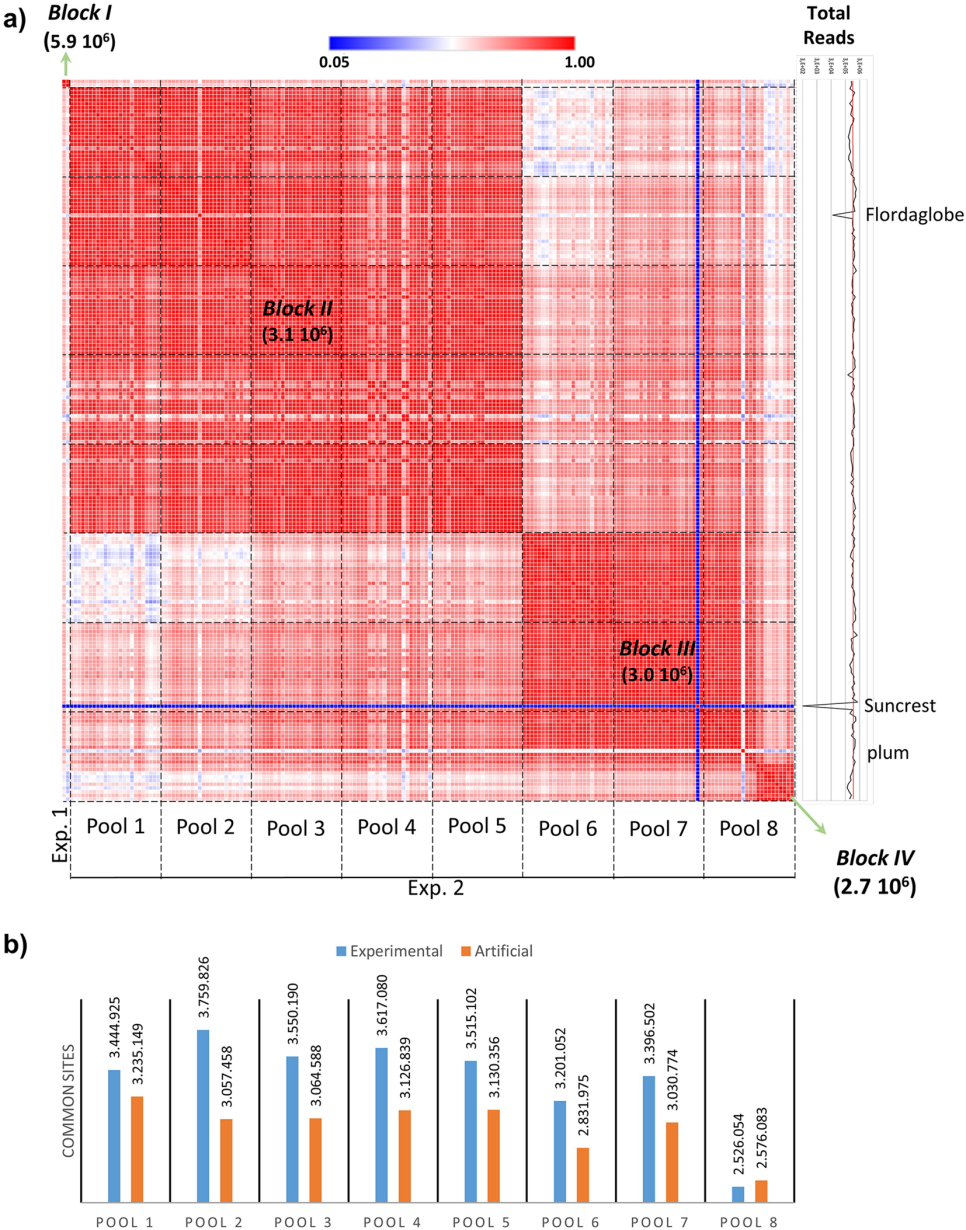
Uniformity of coverage. (**a**) Heatmap of correlations between samples. Color codification of correlation strength is indicated upper the heatmap. At the right, the total number of reads per sample and the mean (indicated with a red line) are shown. Exp. 1, experiment 1; Exp. 2, experiment 2. (**b**) Comparison of the common site observed between samples of the experimental pools (blue) with artificial created pools (red).

The overlapping of genome coverage between samples was analyzed by inspecting the correlations of the reads number mapped in bins among the sample set. A high correlation between two libraries indicates that in average reads fall in the same bins and therefore a high proportion of overlapping genome regions are covered in that two samples. The heatmap revealed four blocks (I, II, III, IV) of highly correlated samples (Fig. 4a). Block I consisted of the two samples used for the fine tuning of the platform (experiment 1). For experiment 2, 8 pools of 24 libraries each were prepared but different grade of similarities between the pools were revealed by the correlation analysis (Fig. 4a). Pools 1–5 formed block II, which indicated similar coverage of genome. Similarly, Pools 6–7 and most of the samples of Pool 8 showed a good coverage between them (Block III). The last 10 samples of pool 8 formed a separate block (IV). As expected, a failing sample (‘Suncrest’) showed a very low correlation (revealed with dark blue in the heatmap) with the rest of the samples. It is important to mention that 296 reads of this sample had been retained after quality check. The plum sample (in Pool 8) also presented low correlations with all peach samples, thus reflecting the genome differences of the two species. Pools 1 and 6 showed the lowest correlation between pools.

The correlations obtained for all pairs of peach samples, except the two failed samples (‘Suncrest’ and ‘Flordaglobe’), ranged from 0.3805 to 0.9798, accumulating around 0.93 (Supplementary Fig. S4). The high correlation within samples of a block translates to a high number of common sites sequenced in all the member of a block. The common sites varied between 2,725,815 for block IV to 5,881,715 for block I (Fig. 4). The failed samples (‘Suncrest’ and ‘Flordaglobe’) and the plum were not considered for common sites determinations.

To assess how the experimental conditions improve the common coverage between samples, we analyzed the number of common sites per pool against 8 artificial pool created in silico by mixing samples from different experimental pools (Supplementary Table S1). The experimental pools showed more common sites than the artificial pools (Fig. 4b), in average 10% more (3,376,341 vs 3,068,163, respectively, α < 0.01, n = 8). Taking into account both experiments, 2,026,509 common sites between the 191 peach samples were scrutinized with the platform described here.

**Figure 5:**
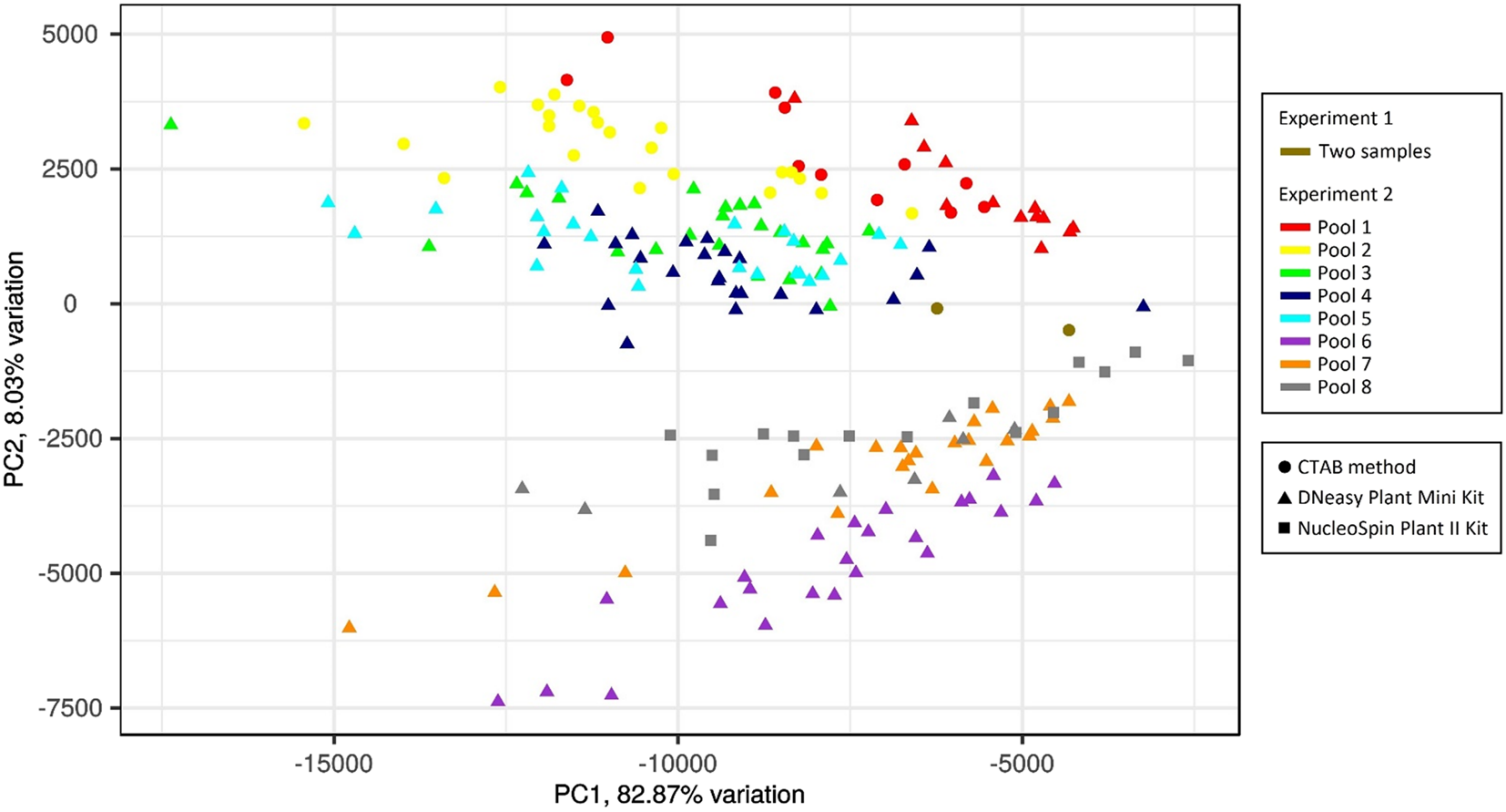
Principal Component Analysis of the number of read mapped on 1 K bins. Samples are codified with different colors according to the batch of analysis (Experiment 1 with two samples, and Experiment 2 with Pool 1–8) and shaped according to the DNA extraction method.

To get further understanding of how experimental conditions affect the overlapping coverage between samples, a PCA was performed with the reads mapped on bins without taking into account the plum and the two failed samples (Fig. 5). A wide proportion of the variance (82.87%) is explained by PC1, which separated samples without an obvious trend (i.e. not according the extraction method or batch of analysis: experiments/pools). The dispersion of samples along the PC1 correlated with the number of reads obtained for each sample (Supplementary Fig. S5). Samples from Blocks I and II are separated from samples from Blocks III and IV according to PC2, which explains 8.03% of the variation. The samples within Block IV are separated by PC5, which accounted for 1.04% of the variance (Supplementary Fig. S6).

### Variant identification and genotyping

To assess the overall power of the platform developed here, the number of variants detected was analyzed. The sequences obtained for all the peach accessions (including rootstocks) comprising 191 genotypes (two from experiment 1 and 189 from experiment 2, in which the plum and the 2 failed libraries were discarded) were analyzed together. The analysis retrieved 113,411 SNP, 13,661 InDel and 2133 SSR polymorphic variants against the peach genome v2.0 in the whole sample set.

**Figure 6:**
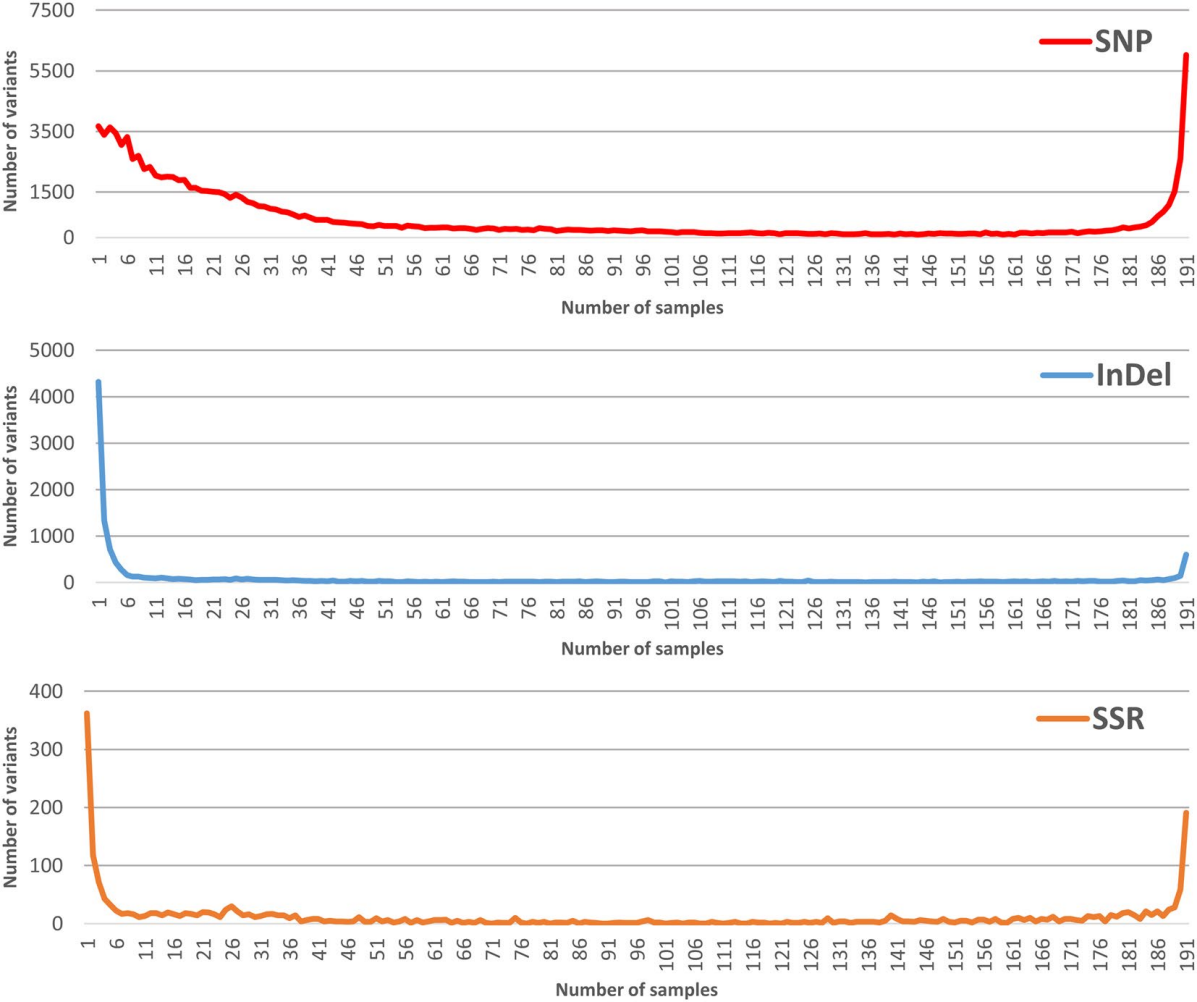
Distribution of the number of variant identified in group of samples. For each kind of variant identified (SNP, InDel and SSR), the number of variants genotyped in 1 to 191 peach samples are shown.

A common drawback of the RADseq platforms is the proportion of missing data between samples^17^. For this reason, we analyzed the number of variants in common along the sample set to assess the platform in this regard (Fig. 6). Taking into account the variants present in only one sample, we identified 3674 SNP, 4318 InDel and 362 SSR. On the other hand, 6028 SNP, 600 InDel and 191 SSR are genotyped in the whole sample set (191 peach accessions). The distribution of the variants followed similar trends, as SNP, InDel and SSR accumulated in few samples or in almost the complete set (191 samples). In the case of SNP, the variants in common dropped slowly until 40 samples and increased sharply from 181 samples to reach a higher number of variants in common in the complete set (6028 SNP). For InDel and SSR, the variants in common dropped sharply at 5 samples and then increased from 189 samples, thus reaching a lower number of variants in common than in that found in one sample. In summary, 50% of the SNP (55,719/113,411) are present in 25 or less samples, whereas, for InDel, the 50% of the variant (6800/13,661) are present in 4 samples or less. For SSR, 4 samples or less account for 25% of the total number of markers identified. On the other hand, 6028 SNP, 600 InDel and 191 SSR are genotyped in the whole sample set (191 peach accessions), thus, identifying one variant for each 297 sites strutted (2,026,509 common site/6819 variant identified).

Several criteria, regarding missing data and minor allele frequency (MAF) accepted, could be taken according the downstream analysis to be conducted^37^. Supplementary Table S3 displays the data sets obtained (for the 191 peach accessions) according to different criteria. In this section, we will restrict our analysis to the data set obtained according to a < 5% of missing data and a minor (MAF) equal to or greater than 1%. This dataset contains 9325 variants, which comprise 7967 SNPs, 980 InDels and 378 SSR. The SNPs are biallelic with 1,521,697 data points (191 × 7967), of which 1.33% are missing data and 14.54% heterozygous positions. The Ts/Tv ratio reached is 1.33, with 225,942 transitions (Ts) and 169,551 transversions (Tv). Regarding the 980 InDels, 91 are triallelic and the remaining 889 are biallelic, with 1071 alternative alleles to the reference genome. The Deletion/Insertion ratio against the peach genome is 0.93, with 515 deletions and 556 insertions. The allele length difference (between the alternative and the reference allele obtained from the peach genome v2.0) was from 1 to 31 bp, with a mode of 1 bp (Supplementary Fig. S7). Almost half of the InDels have a length difference of 1–2 bp (45.75%), 42.67% have a length difference of 3–10 bp, and 11.58% have a difference length of 11–31 bp. With 187,180 (191 × 980) data points for InDel, 1.09% correspond to missing data and 19.23% to heterozygous positions.

In the 378 SSR found in the analysis, 152 are biallelic, 216 triallelic and 10 tetraallelic. The motif length of SSR was from 1 to 6 nt, with 71 (18.78%) mononucleotide, 272 (71.96%) dinucleotide, 22 (5.82%) trinucleotide, 8 (2.12%) tetranucleotide, 3 pentanucleotide (0.79%) and 2 (0.53%) hexanucleotide (Supplementary Fig. S8). Only 3 mononucleotide motifs were found in the analysis: T (39), A (31), and C (1). Regarding the dinucleotide motifs AT (95), AG (92) and CT (63) were the most abundant, whereas GT (13) and AC (9) were the less frequent. For the rest of the motifs, different combinations of nucleotides occurred in low proportion (Supplementary Fig. S8). In addition, following Webber's criterion^38^, 12 of the SSR are imperfect and the remaining 366 are perfect. In the 72,198 (191 × 387) data points for SSR, 1.25% has missing data and 30.38% heterozygous positions.

The potential of the platform was assessed for functional variant identification by analyzing the predicted effect of the markers on the peach genome. The 9325 identified variants may cause 42,509 putative effects, according to the analysis. The high number of predicted effects could be due to the presence of multiple transcripts for a gene and to the fact that the analysis takes into account the effect of each one. Moreover, some genes overlap, so a single variant could affect multiple transcripts on multiple genes, with different effects^35^. Our study identified 89 genes of high impact, 1532 of moderate impact, 2341 of low impact, and 38,553 modifiers (Supplementary Fig. S9). Regarding the affected genomic region, the most affected areas are the downstream (up to 5 kb downstream of polyA addition site), upstream (up to 5 kb upstream of the transcription start site) and intronic region, with an impact of 32%, 26% and 19%, respectively (Supplementary Fig. S10). Importantly, although in lower proportion, many areas of interest were affected. For example, 3510 (8.25%), 1471 (3.46%) and 996 effects (2.34%) take place in the exonic region, 3′ UTR and 5′ UTR, respectively (Supplementary Fig. S10). From these data, we determined 1946 synonymous substitutions and 1497 nonsynonymous substitutions.

**Figure 7:**
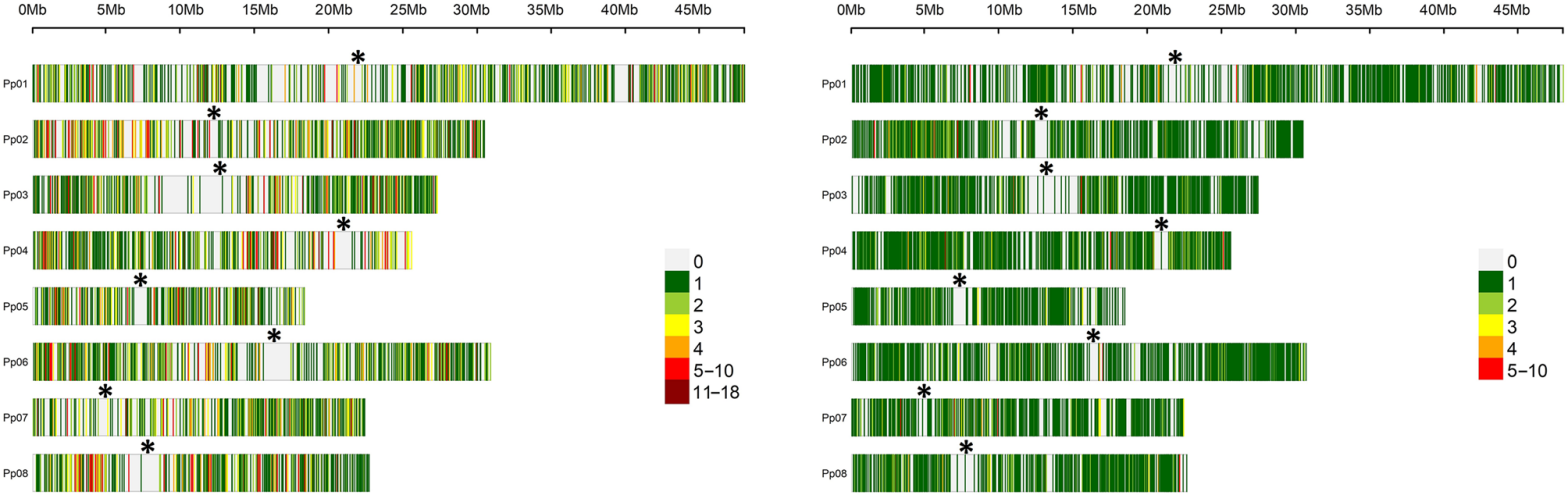
Density of SNPs along chromosomes. Number of SNPs within 1 Kb window size for the 7967 SNPs obtained with the platform developed (left) and the Ilumina 9 K SNP array (Verde et al.^5^, right) are shown. Vertical bar at the corners indicates the color assigned to the SNP number per 1 Kb window. The asterisks (*) indicate the putative location of centromeres.

The distribution of variants along the peach genome was analyzed and compared to the 9K SNP Infinium II (Fig. 7 and Supplementary Fig. S11). The SNP covered all the 8 peach chromosomes, with the exception of the region near to the centromeres, as the case of the SNP array. The platforms shared only 133 SNPs in common. In our platform, most of the 1 kb-bin covered has 1 SNP, although the platform allowed the discovery of some hot spot of density, for example at the top of Chromosome 2 (Pp02) and the bottom of Chromosome 4 (Pp04) (Fig. 7). Despite covering less proportion of the genome, the InDel and SSR were detected in all chromosomes (Supplementary Fig. S11).

## Discussion

### A sequencing yield of 1 M of 2 × 250 pair-end read is suitable for the analysis of peach genome under the conditions described

The digestion with the enzymes MboI and PstI produces 63,730 putative loci in the region of 300–400 bp according to in silico simulation; which was the highest number of loci for the conditions analyzed (Supplementary Table S2). The experimental analysis supported the simulated predictions, since the double restriction with PstI/MboI produced the highest fragment population in the 300–400 bp region (Fig. 1). Theoretically, these conditions would generate between 19.119 × 10^6^ pb (63,730 × 300 bp) to 25.492 × 10^6^ bp (63,730 × 400 bp) of DNA. We set an average sequencing yield of 250 × 10^6^ pb [1 × 10^6^ of paired-end (2 × 250 bp) reads] per sample to ensure at least 10× depth coverage. Accordingly, we obtained an average of 1.04 × 10^6^ of paired-end (2 × 250 bp) reads and a 15 × depth coverage for the 191 peach samples. This result indicates that our design was appropriate. In spite of setting the condition to obtain 1 × 10^6^ reads for each sample, we detected a dispersion of sequencing with most of the samples in the interval of 1.0 × 10^6^ ± 600,000 reads (Fig. 2, Supplementary Fig. S2).

Since the breadth of coverage increase until 1.5 × 10^6^ reads, it seems convenient to increase the expected reads to this value to enhance the chances of getting at least 1 × 10^6^ reads for each sample under the conditions described in this work. We anticipate that under this recommendation, a higher number of common site between samples will be reached. Recently, another study has reported the use of the GBS protocol based on double restriction with PstI/MspI that we consider in the fine-tuning of the platform. In that study, the researchers has genotyped 53 *Prunus rootstocks*^21^ with a higher sequence deep (average of 11 M/sample) and shorter reads (2 × 125  pb) and reported a 45 K SNP data set. It is important to mention that they have included only one peach (*P. persica*) accession and 32 interspecific hybrids of *P. persica* and other P*runus* species in the study. This higher number of variants could be attributable to the inclusion of different species backgrounds in the analysis. For our genomic data, the joint analysis of the 191 peaches with a plum genotype (*P. salicina*) allowed the identification of 161,977 SNP in total and gave rise to a data set of 45,133 SNP present at least in 95% of the samples, and a data set of 23,448 SNP present in all the samples (data not showed). It will be of great interest to apply that protocol in a peach germplasm collection to compare to the platform described here.

### Uniform experimental conditions enhance genome representation by increasing the number of the analyzed loci in common

The peach genome was uniformly covered by reads with the exception of the chromosome regions (Fig. 3) that are predicted to harbor the centromeric structures^4^. Since that centromeres are mainly composed of highly repetitive and methylated sequences is expected that a low frequency of restrictions take place and therefore large DNA fragments are produced during reduced representation, which are discarded at the selection size step. The distribution of reads along chromosomes pointed a region, Pp01-14,777,945–14,778,945, that accumulated an unusual high number of reads that was analyzed to get a deep understanding. A blast analysis of the region identified sequences with high homology to mitochondrial sequences (Supplementary Fig. S3). This may be explained by methodological flaws. Indeed, DNA from that organelle may have been captured in our experiment and, due to high relative levels compared to other loci, an elevated number of reads may have been mapped to a genome region with homology to mitochondrial sequences. Further experiments are needed in order to disclose if this sequence is repeated in both mitochondrial and nuclear genomes or the accumulation is due to an artifact generating by a misassembling of the peach genome v2.0 at that region.

We took advantage of the diverse sample set to assess how experimental conditions affect the overlapping genome coverage between samples by a combination of multivariate analyses (Figs. 4a, 5 and Supplementary Fig. S6). In the case of the experiment 1, we expected that the samples showed similar coverage between them, in comparison to the rest of the sample set, and that they clustered together (Fig. 4a). This speculation was due to the fact that the library preparation and the selection step were different in both experiment. The selection step was performed manually in experiment 1, while being automatic in experiment 2. Nevertheless, three groups of samples (Blocks II, III and IV) were obtained in the analysis for experiment 2. Because the construction of the libraries in the pools were performed sequentially (starting with pool 1 and ending with pool 8), we hypothesized that an unidentified experimental change (e.g. trademark of reactive or minor equipment setting) occurred between pool 5 and 6 and that this in turn could be the reason for the formation of the main blocks of samples (II and III). In accordance with this view, PC2 (which accounts for 8.03% of the variance) separated the samples at this point (Fig. 5). The case of pool 8, in which few samples clustered in a different block (IV, Fig. 4a), is particular and may be due to a technical bias. According to the PCA, these samples were separated within PC5 that accounted for 1.04% of the variance. Nevertheless, most of the variance was represented by PC1 (82.87%), with the samples dispersed along this axis according the sequencing yield (Fig. 5, Supplementary Fig. S5). Therefore, most of the variation in our study seems to be due to uncontrolled experimental conditions that resulted in an unequal amount of the library material, thus leading to the dispersion of the samples. Moreover, according to the PCA, there is no clear association between the samples extracted with CTAB method or the two commercial kits used (Fig. 5). Altogether, and taking into consideration that the extraction method does not affect the number of obtained reads, we suggest that the platform is robust regarding the purity of the starting DNA.

Is expected that as more genome site in common within the sample set are analyzed, more variant could be obtained. We thus assessed how the reduction in the overlapping representation of the genome analyzed is translated to less sites in common (Fig. 4). In average, the artificial pools have 10% less sites in common than those of the actual experimental pools which is an estimation of the effect of the experimental conditions in the reduction of the number of loci in common. This could be attributed to the library construction procedure and/or the selection step, since these process are developed in batch.

We observed a dispersion on the reads yield within the sample set (Supplementary Fig. S2) with 28.14% of VC. The VC observed was lower than that found for the same platform applied in *E. dunnii* (39%) and other ddRADseq protocols (42–47%, Aguirre et al.^23^ and references within). No association was detected between read yield and experimental pools (Fig. 4); and the variability due to the dispersion of the reads yield was distributed in the overall experiment (Supplementary Fig. S5).

### The platform developed is suitable for the identification and high-throughput genotyping of peach variants

In this work, we presented a NGS-based platform that allowed the identification of 113,411 SNP, 13,661 InDel and 2133 SSR in a set of 191 peach accessions. By applying an ApeKI-GBS protocol, Thurow et al.^20^ identified 93,353 SNP in 217 peach genotypes. The total number of SNP are comparable, although different germplasm, protocols (ddRAD-Seq vs ApeKI-GBS), sequencing technologies (2 × 250 pb vs 1 × 100 bp) and read depth (1 M/sample; 1.45 M/sample) were used in both studies^20^. Considering MAF > 0.05 and 25% of accepted missing data, the obtained data set was lower (6929 SNP vs 18,373 SNP, Supplementary Table S3). Nevertheless, Thurow et al.^20^ did not report the dataset obtained considering a lower missing data accepted (i.e. 5%). In addition, if the germplasm under study are highly different to that of the peach reference genome, more variants are expected to be discovered.

Guajardo et al.^21^ reported a dataset of 45 K SNP (MAF > 5%; missing data 5%). Unfortunately, the dataset are not comparable with our results, since the analyzed germplasm includes interspecific hybrids between *Prunus persica* and other *Prunus* (*P. dulcis*, *P. cerasifera* and *P. davidiana*) and other hybrids and species of *Prunus* from a different subgenus (*P. avium*, *P. tomentosa*, *P. mahaleb*, *P. cerasifera*, *P. besseyl*, and *P. salicina*). In our study, we expected that three accessions have a proportion of *P. davidiana* in their genomes (Supplementary Table S1). Although the analysis is not presented here, if these accessions are not considered for variant calling, the total number of variants (110,671 SNP, 13,246 InDel and 2114 SSR) as well as the selected data set (7,390 SNP, 946 InDel and 355 SSR, MAF > 1%; missing data 5%) are similar to the reported in Supplementary Table S3.

Other option for peach genotyping is the use of SNP array platforms. RosBREED consortia announced the release of novel 16 K and 18 K arrays for peach (https://www.rosaceae.org/analysis/267). Up to date there is not published result using these platforms. For this reason, we compared our data to the original 9 K SNP Infinium II array developed by Verde et al.^5^ and used in several other studies. The SNPs obtained with the ddRADSeq platform covered all the peach genome (Fig. 7) with an overall similar density, with the exception of the region near to the centromeres. This finding was actually expected, since less reads occurred at these regions (Fig. 3). The 9 K SNP array is also less dense at the centromeres, but has more uniform density of markers. The latter is in line with the fact that the array was designed for an even distribution the SNP. Our platform identified genomic regions with higher density of markers (Fig. 7), thus suggesting the existence of hot spots of highly variable regions within the peach genome. After genotyping 1576 peach varieties with the 9 K array, a data set of 4271 SNP was obtained considering MAF > 5% and 5% of missing data^14^. Although the number of genotypes is not comparable (191 vs 1576), the platform described here reached similar number of SNP for a MAF > 5% and 5% of missing data (4627 SNP, Supplementary Table S3) but different loci are scrutinized since only 133 SNP were found in common between the platforms. The sequencing data provide the flexibility to use different data sets according to the studies to be conducted. For example, for genetic studies of the germplasm collections or GWAS, a data set with lower percentage of missing data (e.g. 5%) and variant of a minimum MAF (e.g. 1%) will be desired. For that example, if a less restricted criterion is taken (MAF > 1% and 25% of missing data), the platform provides a dataset of 12 K variants (Supplementary Table S3). Nevertheless, for other purposes like the identification of polymorphic marker between two parental genotypes or for the analysis of a subset of samples, the platform provides data sets with values above 6 K (since 6028 variants are present in the 191 accessions).

Apart from SNPs, the platform allowed the identification of 13,661 InDel and 2133 SSR that could be useful for other applications, such as pedigree identification and construction of genetic maps, because of the polymorphic nature of these variants. Even in the case of the reduced data set of 9 K, the inclusion of this type of variant improves the chance to identify causal loci since most of them cause frameshift if they are present in exons. To assess the platform in this regard, we annotated the 9 K data set (including SNP, InDel and SSR) according to the predicted effects on the peach genome. Almost 10% of the effects detected (9.32%) are predicted to have a significant impact (Supplementary Fig. S9). Strikingly, most of the effects are in the surrounding of genes (upstream and downstream). This could be due to the fact that methylation sensitive enzymes were used for genome complexity reduction, thus avoiding repetitive non coding regions to be sampled. However, a more general feature of the peach genome could not be discarded (e.g. since the peach genome is compact is expected that statistically a region will be near a gene). Regarding the impacts that fall within a gene, most of them are in the intron regions. This is probably due to the fact that the introns accumulate more mutations than the coding regions.

In summary, the novel ddRADseq platform for peach described here allows the identification and genotyping of a wide number of variants. The total number of genotyped SNP, taking into account the accepted threshold of MAF and missing data, are comparable with other technologies used in peach so far. However, the platform that we described has the advantage of genotyping InDel and SSR as well. The datasets described here were used to conduct genomic studies such as GWAS and cultivar identification that will be presented elsewhere since are beyond the scope of this article.

## Conclusions

In this study, we performed a fine-tuning of ddRADseq protocol dedicated for peach. The platform based on NGS technology allowed a high-throughput variant identification and genotyping of a wide peach collection. Factors affecting the overlapping genomic regions were discovered and their putative effects estimated on loci in common was scrutinized. This was translated into a percentage of missing data, the main limitation of RADseq/GBS technologies. A detailed description of the platform and the comparison with other genotyping methods described for peach suggested that the platform is suitable for conducting genomic based breeding in peach.

## Supplementary Information

Supplementary Information 1.

Supplementary Information 2.
