# Supplementary material for: Fine-tuning the performance of ddRAD-seq in the peach genome
Source: Sci Rep. 2021 Mar 18;11:6298. doi: 10.1038/s41598-021-85815-0 (PMC7973760; doi:10.1038/s41598-021-85815-0)
Supplement: Supplementary file 1 — Supplementary Information 1. [file 41598_2021_85815_MOESM1_ESM.pdf]

Supplementary Figures

Fine-tuning the performance of ddRAD-seq in the peach genome

Maximiliano Martín Aballay[1], Natalia Cristina Aguirre[2], Carla Valeria Filippi[2], Gabriel Hugo Valentini[1] and Gerardo Sánchez[1*]

[1] Laboratorio de Biotecnología. Estación Experimental Agropecuaria (EEA) San Pedro, INTA, Ruta N°9, Km 170. San Pedro (2930). Argentina.

[2] Instituto de Agrobiotecnología y Biología Molecular–IABiMo–INTA-CONICET, Instituto de Biotecnología, Centro de Investigaciones en Ciencias Veterinarias y Agronómicas, Instituto Nacional de Tecnología Agropecuaria, Hurlingham (1686), Argentina

*corresponding author (sanchez.gerardo@inta.gob.ar)

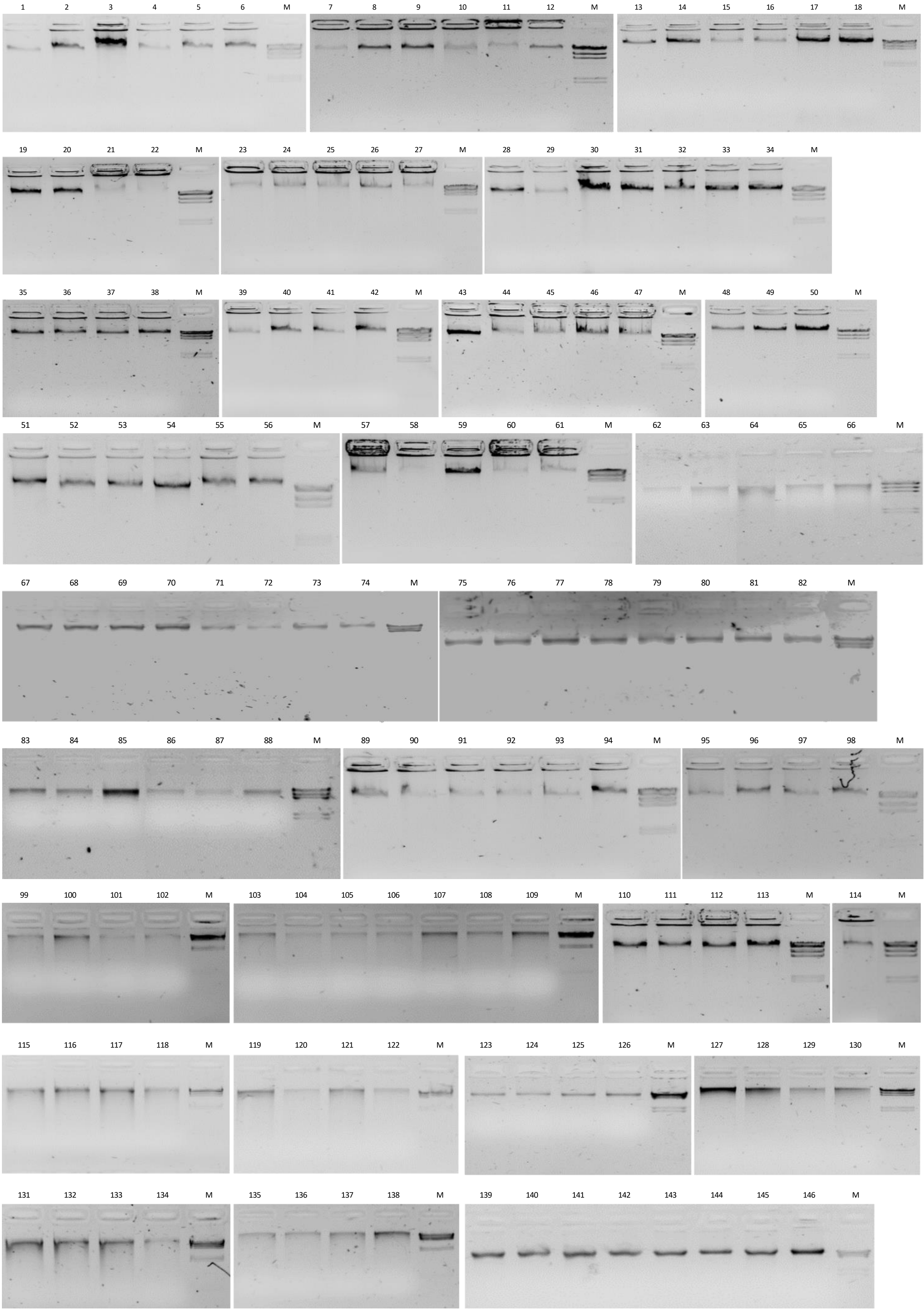
1 2 3 4 5 6 M 7 8 9 10 11 12 M 13 14 15 16 17 18 M
19 20 21 22 M 23 24 25 26 27 M 28 29 30 31 32 33 34 M
35 36 37 38 M 39 40 41 42 M 43 44 45 46 47 M 48 49 50 M
51 52 53 54 55 56 M 57 58 59 60 61 M 62 63 64 65 66 M
67 68 69 70 71 72 73 74 M 75 76 77 78 79 80 81 82 M
83 84 85 86 87 88 M 89 90 91 92 93 94 M 95 96 97 98 M
99 100 101 102 M 103 104 105 106 107 108 109 M 110 111 112 113 M 114 M
115 116 117 118 M 119 120 121 122 M 123 124 125 126 M 127 128 129 130 M
131 132 133 134 M 135 136 137 138 M 139 140 141 142 143 144 145 146 M

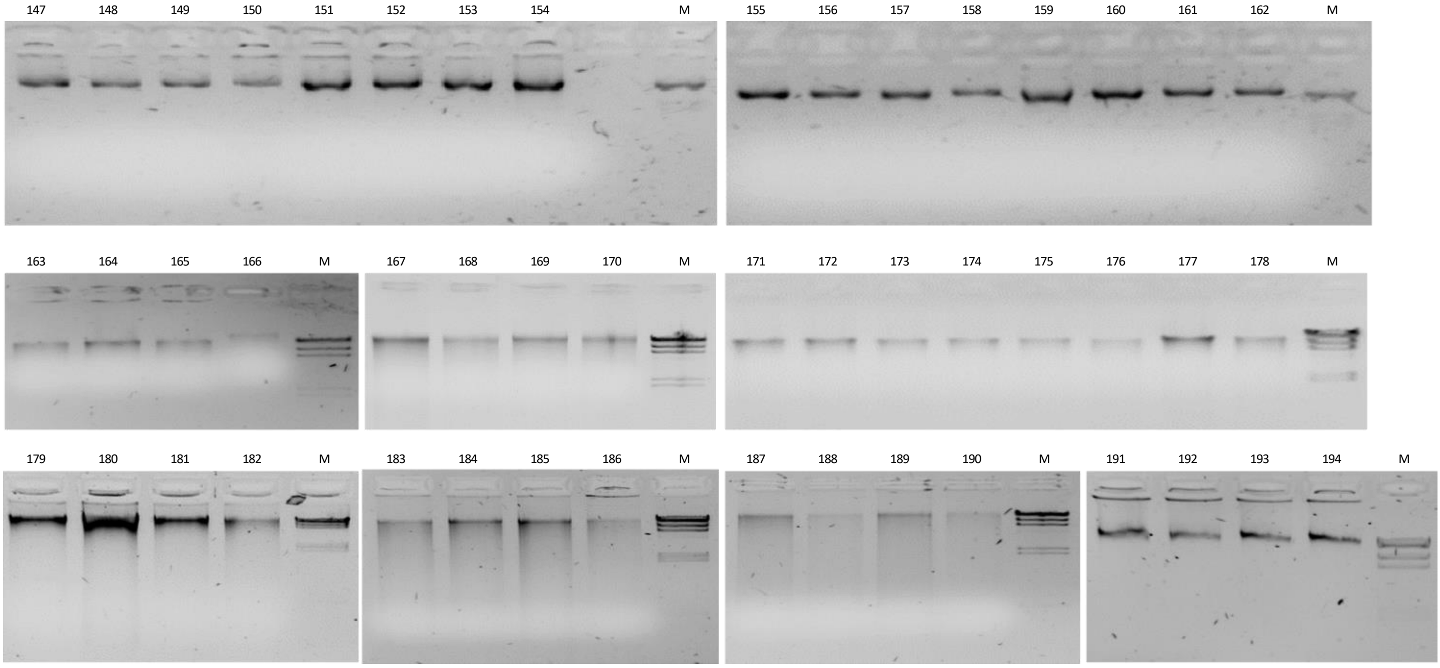

Supplementary Fig. S1. Agarose gel electrophoresis analysis. Quality verification of genomic DNA extraction from 194 accessions. The numbers at the top of images are according to the numbering of Supplementary Table S1, and the molecular size marker(Lambda Hind III) is indicated with letter “M” in the right side of each gel.

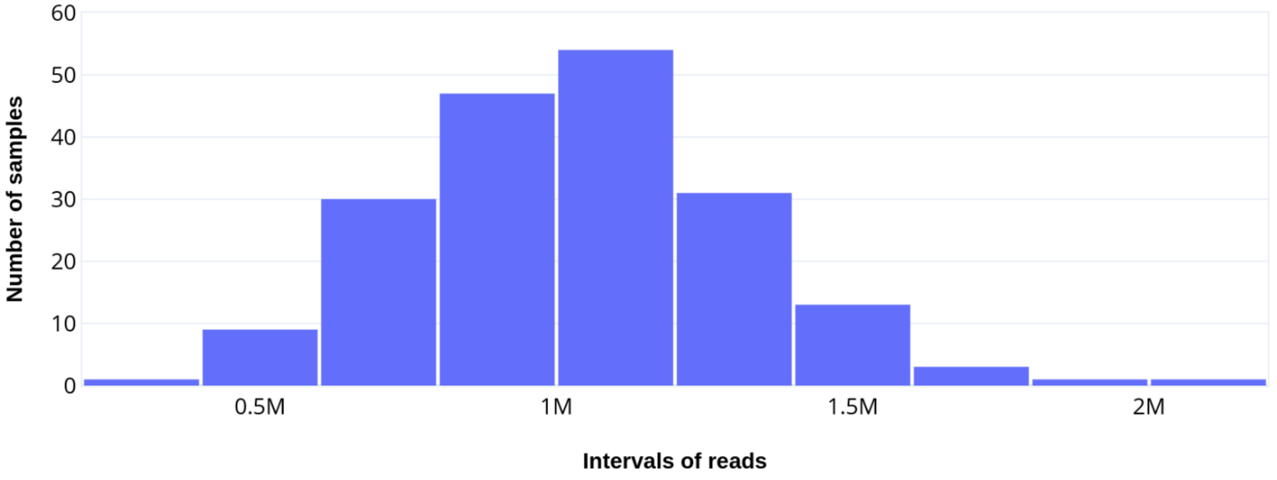

Supplementary Fig. S2. Histogram of reads distribution. The number of samples present in 200,000-reads intervals is presented.

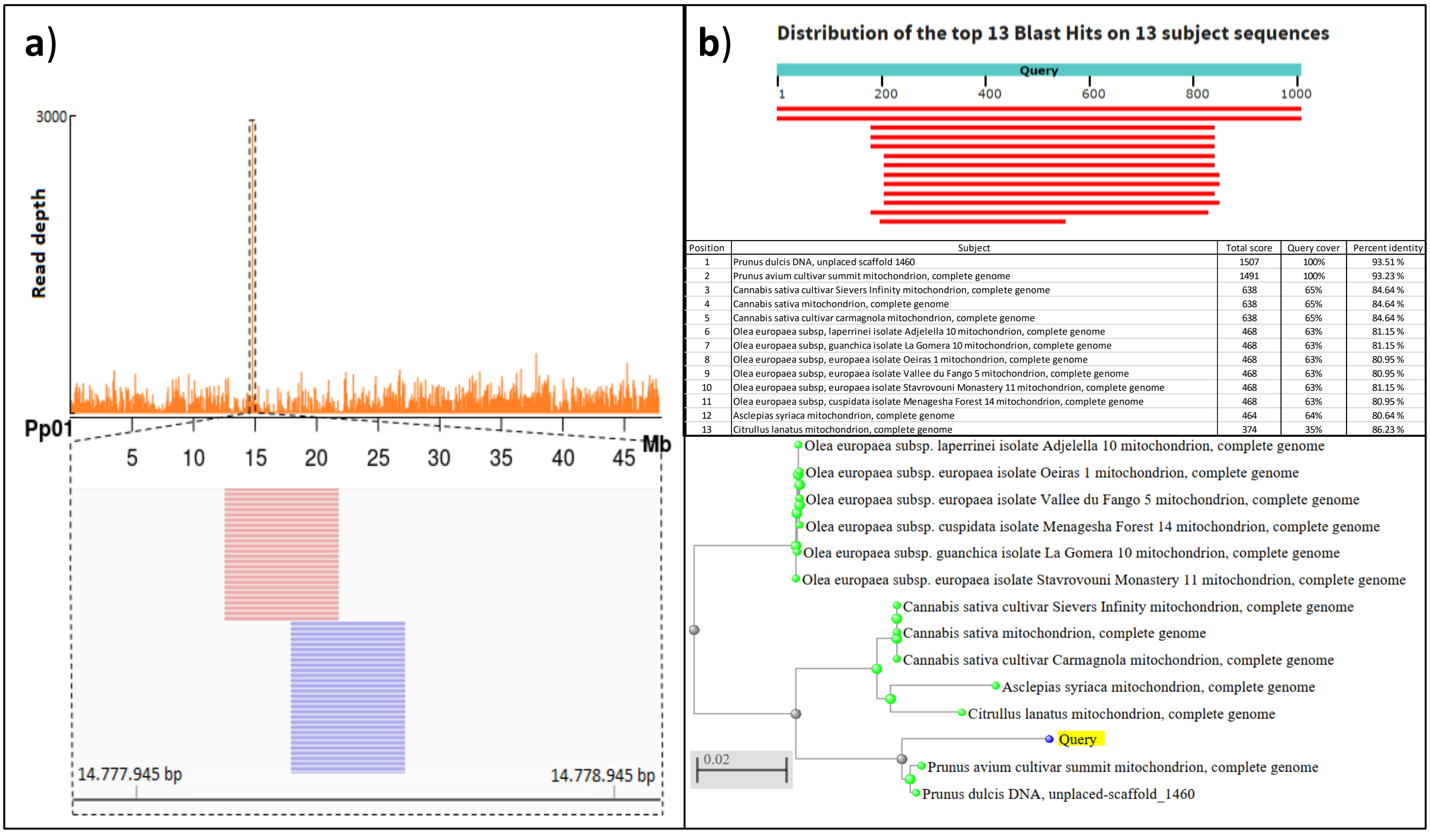

Supplementary Fig. S3. Analysis of the bin Pp01-14777945-14778945. a) The accumulation of reads at position 14,777,945-14,778,945 for all the samples (up) and the accumulation of reads mapped for an aleatory sample (down). **b**) Blast analysis using the sequence of the bin Pp01-14777945-14778945 as query. The sequences with higher identity and the corresponding phylogenetic tree are shown.

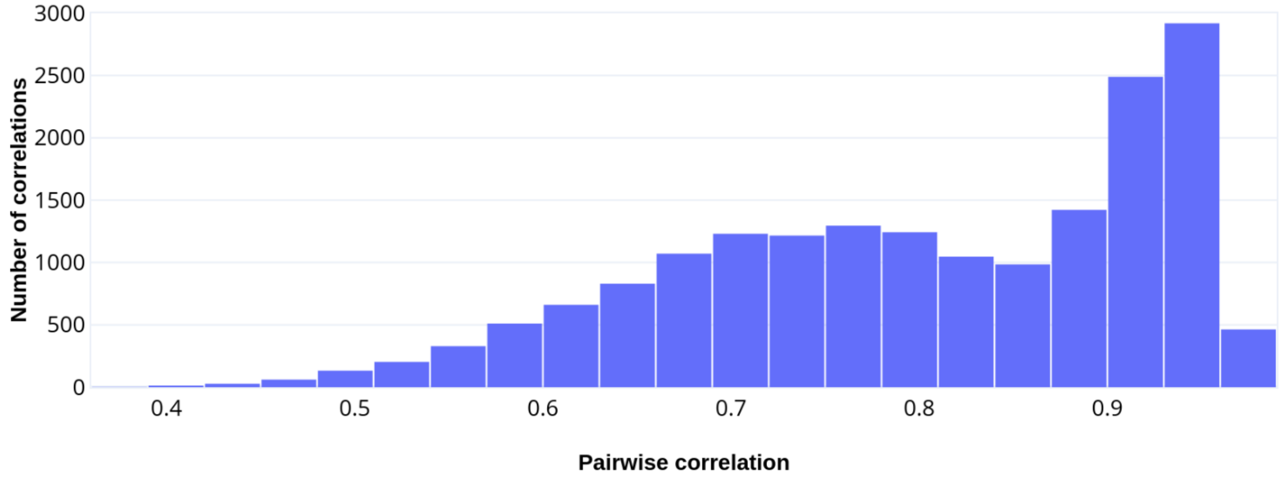

Supplementary Fig. S4. Histogram of pairwise correlations. Distribution of all pairwise correlations among the peach samples, where each bar represents an interval of 0.03.

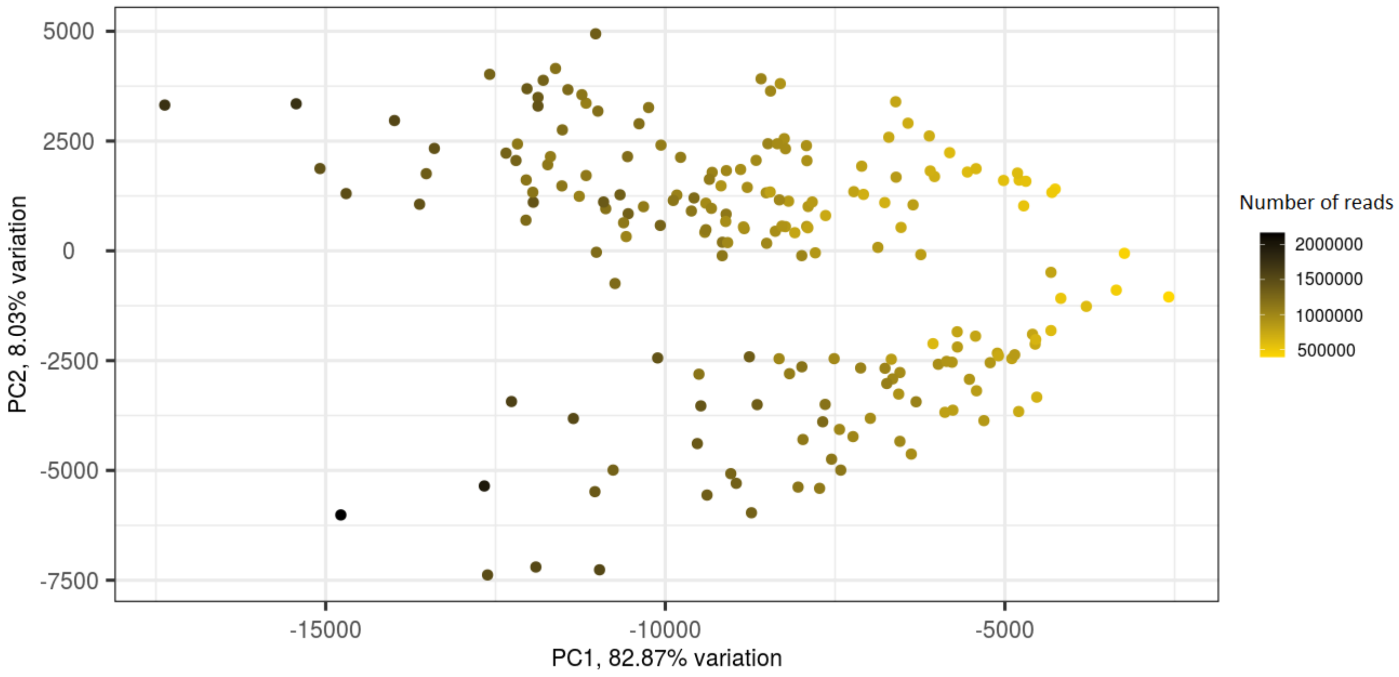

Supplementary Fig. S5. Dispersion of samples along PC1. The color of the samples is based on the number of reads of each one, according to the scale of the vertical bar.

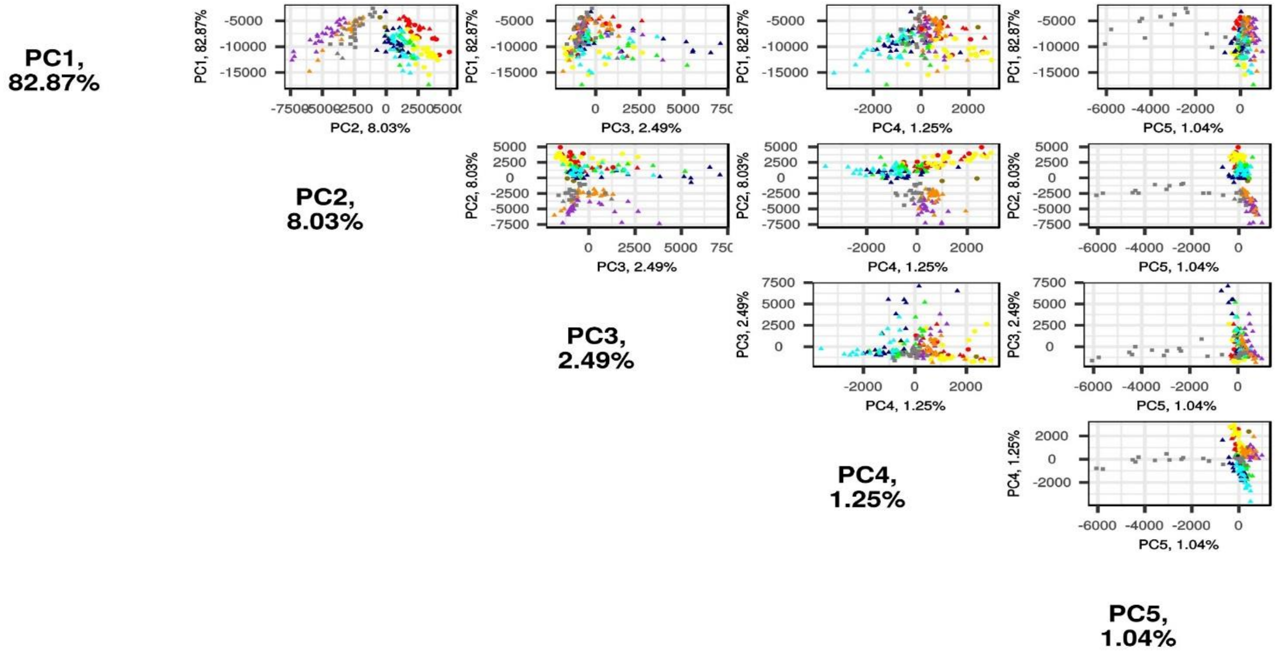

Supplementary Fig. S6. Analysis of the combination of principal components 1-5. The samples are codified with colors (experiment/pools) and shapes (DNA extraction methods) as the used in Fig. 5. The PC5 separate samples belonging to block IV. The PCs 1-5 explain 97.68% of the variance.

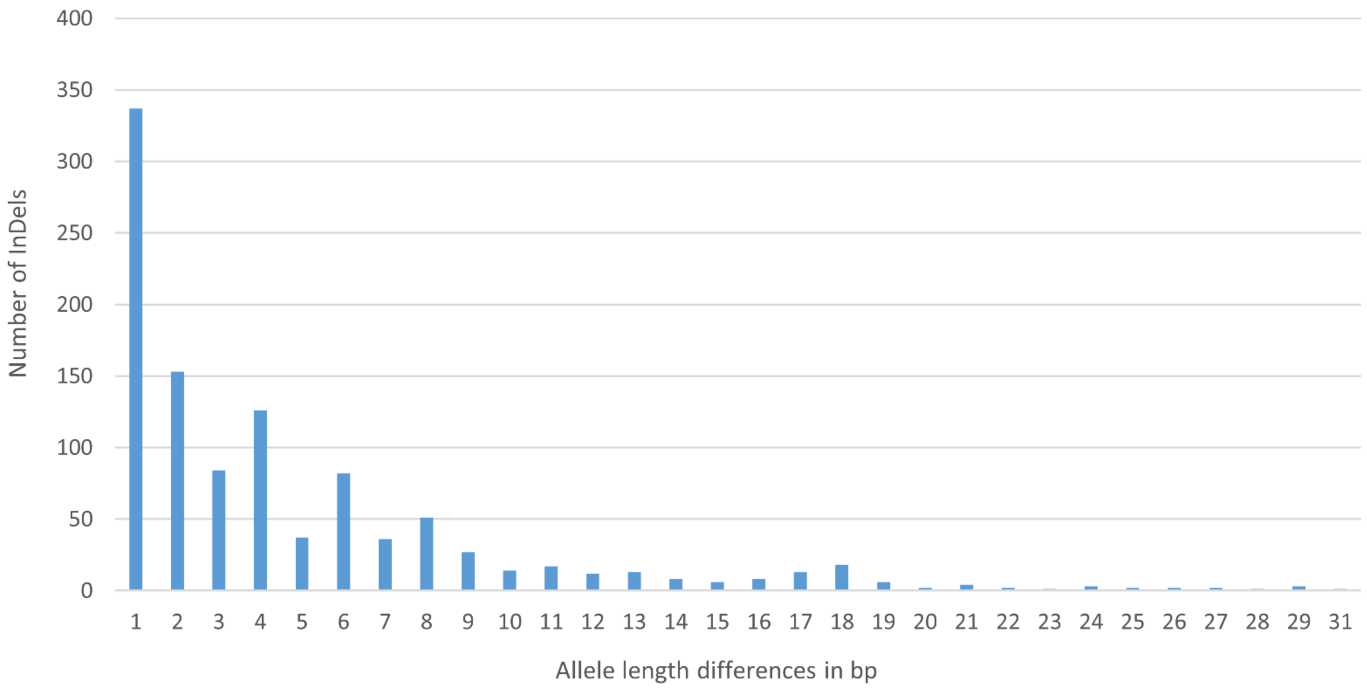

Supplementary Fig. S7. Allele length of InDels. The number of alleles with differences in length with respect to the reference genome are shown.

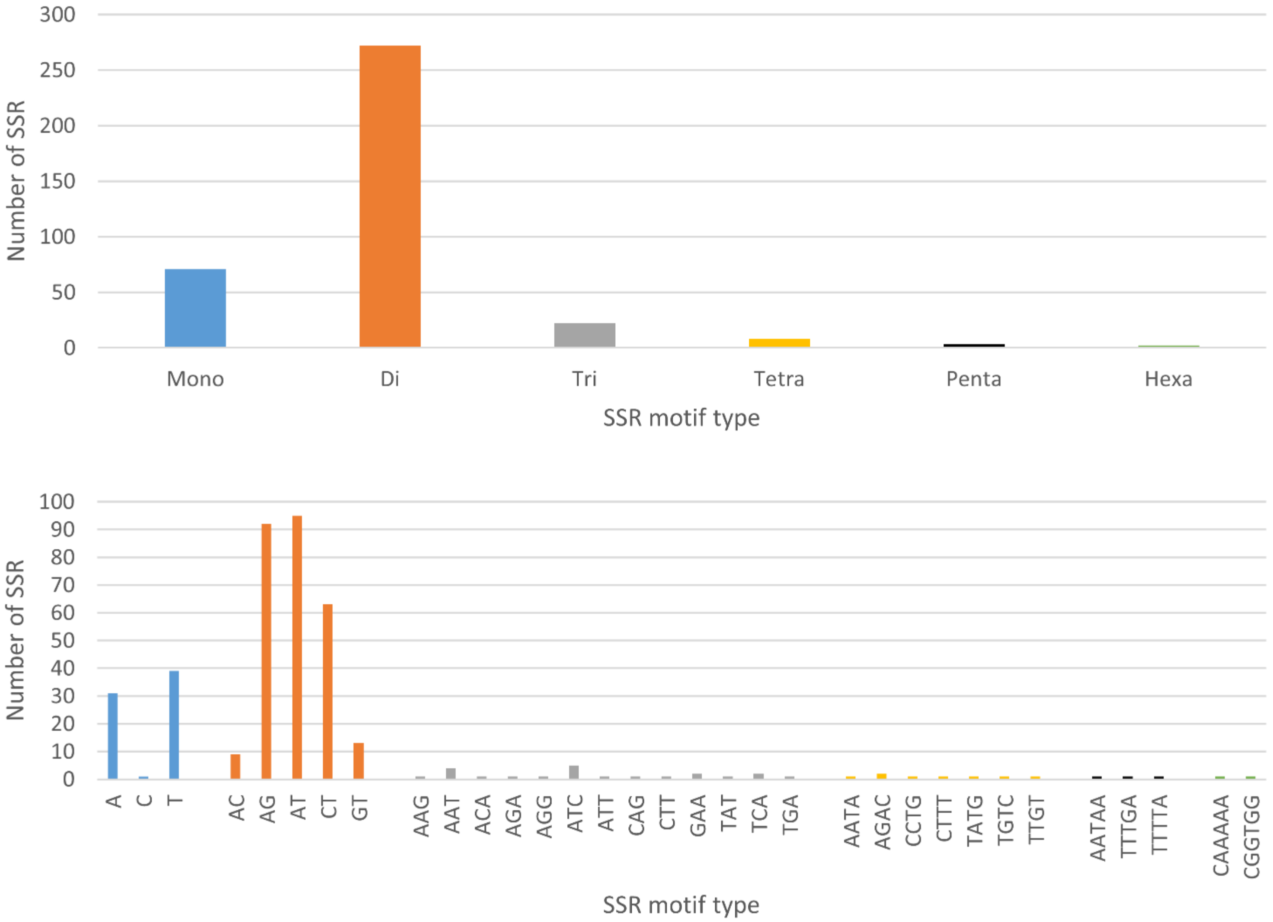

Supplementary Fig. S8. SSR description. The number of SSR according to the repetitive motif (up), and the composition of this motif (down) are shown.

Impact	Meaning	Genomic effects
HIGH	The variant is assumed to have high (disruptive) impact in the protein, probably causing protein truncation, loss of function or triggering nonsense mediated decay.	Splice site acceptor<br>Splice site donor<br>Start lost<br>Exon deleted<br>Frame shift<br>Stop gained<br>Stop lost
MODERATE	A non-disruptive variant that might change protein effectiveness.	Non synonymous coding<br>Codon change<br>Codon insertion<br>Codon change plus codon insertion<br>Codon deletion<br>Codon change plus codon deletion<br>UTR 5' deleted<br>UTR 3' deleted
LOW	Assumed to be mostly harmless or unlikely to change protein behavior.	Synonymous start<br>Non synonymous start<br>Start gained<br>Synonymous coding<br>Synonymous stop<br>Non synonymous stop
MODIFIER	Usually non-coding variants or variants affecting non-coding genes, where predictions are difficult or there is no evidence of impact.	Exon<br>Intron conserved<br>UTR 5' region<br>UTR 3' region<br>Downstream<br>Intragenic<br>Intergenic<br>Intergenic conserved<br>Upstream<br>Regulation<br>Intron

Supplementary Fig. S9. Categories of impact effect and meanings. The impacts were classified according the predicted genomic effects in Cingolani et al. [35].

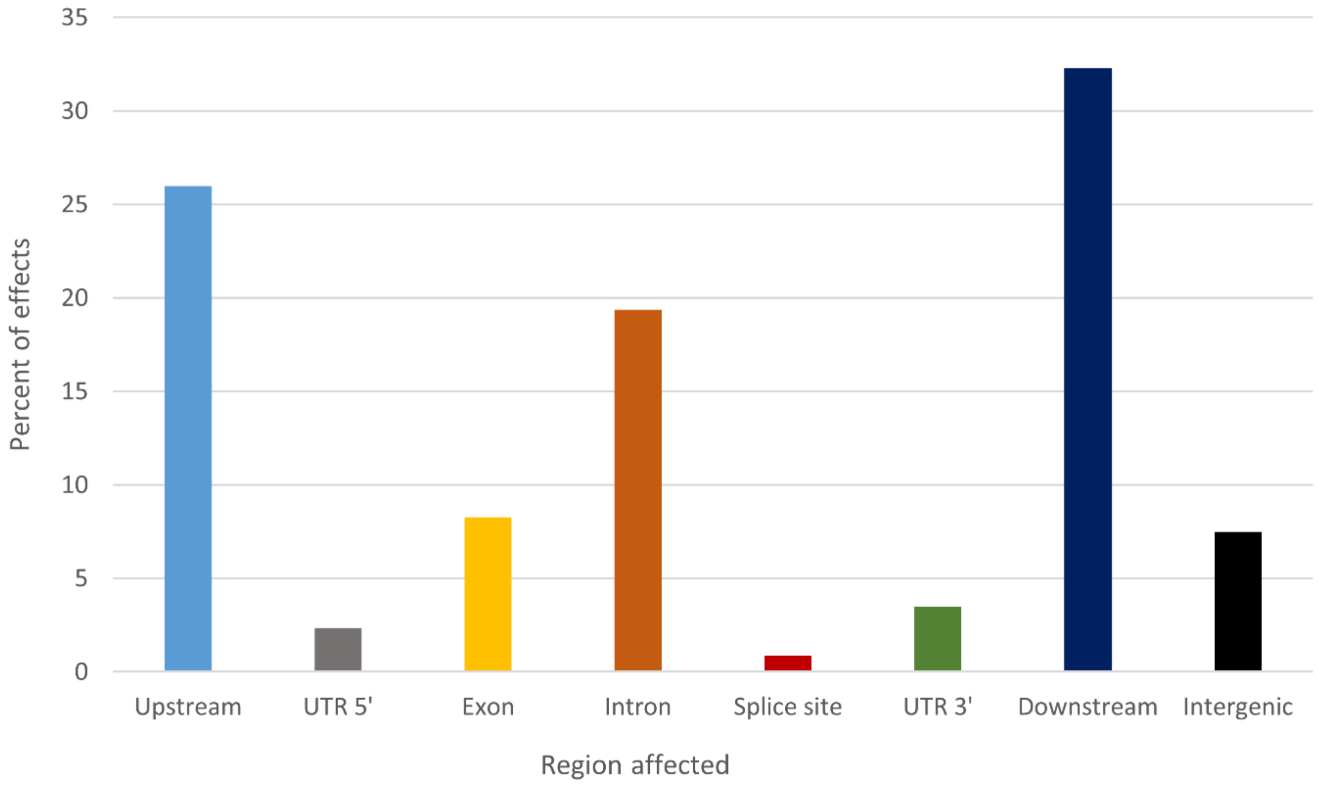

Supplementary Fig. S10. Distribution of effects by region affected. The percentage of the impact according the different regions affected are shown with colored bars.

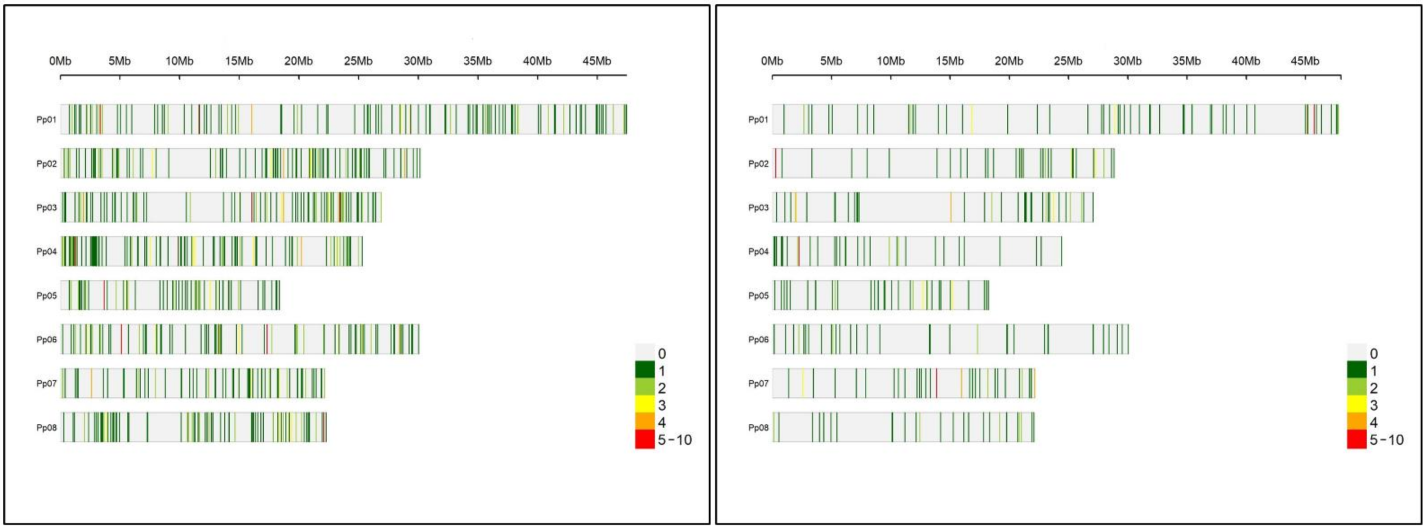

Supplementary Fig. S11. Density of InDels (left) and SSR (right) along chromosomes. Number of markers within 1 Kb windows size, for the 980 InDels and 378 SSR obtained with the platform developed. Vertical bar at the corners indicates the color assigned to the markers number per 1 Kb window.
